# Gold Nanoparticles Contact with Cancer Cell: A Brief Update

**DOI:** 10.3390/ijms23147683

**Published:** 2022-07-12

**Authors:** Nora Bloise, Silvia Strada, Giacomo Dacarro, Livia Visai

**Affiliations:** 1 Department of Molecular Medicine, Centre for Health Technologies (CHT), INSTM UdR of Pavia, University of Pavia, 27100 Pavia, Italy; strada.silvia96@gmail.com (S.S.); livia.visai@unipv.it (L.V.); 2 Medicina Clinica-Specialistica, UOR5 Laboratorio di Nanotecnologie, ICS Maugeri, IRCCS, 27100 Pavia, Italy; 3 Department of Chemistry, University of Pavia, 27100 Pavia, Italy; giacomo.dacarro@unipv.it

**Keywords:** gold nanoparticles, cancer cells, targeting molecules, protein corona, tumor microenvironment, nanoproteomics

## Abstract

The fine-tuning of the physicochemical properties of gold nanoparticles has facilitated the rapid development of multifunctional gold-based nanomaterials with diagnostic, therapeutic, and therapeutic applications. Work on gold nanoparticles is increasingly focusing on their cancer application. This review provides a summary of the main biological effects exerted by gold nanoparticles on cancer cells and highlights some critical factors involved in the interaction process (protein corona, tumor microenvironment, surface functionalization). The review also contains a brief discussion of the application of gold nanoparticles in target discovery.

## 1. Introduction

A significant amount of work has been carried out on the synthesis and applications of metals nanomaterials [1]. At a nano-level, the optical and electronic properties of gold nanoparticles (AuNPs) are unique and tunable by changing the size, shape, surface chemistry, or aggregation state [2]. AuNPs can rapidly and efficiently absorb visible, UV, and NIR light and release energy as heat [3]. AuNPs also possess low toxicity, high stability, simple synthesis, and conjugation with specific biomolecules [4,5]. The fascinating physicochemical features of AuNPs make them a resourceful nanoplatform for outstanding biomedical applications, such as in the areas of photothermal- and immuno-therapies [6], radiotherapy [7], and drug delivery [3]. The ability to functionalize AuNPs surface with targeting moieties allows for their selective delivery to cancer sites with exciting potential. In the last years, numerous publications and clinical trials have emphasized the promise of AuNPs-dependent cancer diagnosis and therapy, with many reviews on the topic [4,8]. Numerous chemotherapeutic drugs—including Paclitaxel (PTX), 5-fluorouracil (5-FU) and Gemcitabine (GMC)—have been conjugated with AuNPs to reduce doses and, consequently, the side effects of treatment [9]. It has also been established that Methotrexate (MTX) or Doxorubicin (DOX) conjugated-AuNPs demonstrate higher levels of cytotoxicity and better accumulation in tumor cells than free MTX or DOX [10,11]. It remains the case, however, and as recently reviewed by Lopes et al. [8], that despite the high number of gold-based nanomaterials developed there are persistent translation challenges with AuNPs. In fact, although AuNPs show potentially useful properties in many preclinical studies, there are very few clinical trials assessing the use of AuNPs for cancer diagnostics and therapy, and, to date, no preparations containing AuNPs have been used effectively in clinical practice [12,13,14]. CYT-6091 (Aurimune) was the first product in clinical trials using AuNPs functionalized with the recombinant human tumor necrosis factor alpha (rhTNFα) for patients with advanced solid tumors (ClinicalTrials.gov Identifier: NCT00356980, NCT00436410). AuroShell, a silica–gold core–shell nanoparticle, is another example of AuNPs undergoing clinical trials for thermal ablation of localized tumors (NCT00848042, NCT01679470). Another formulation under clinical investigation is a NU-0129, a spherical nucleic acid (SNA) AuNPs, as a treatment for patients with recurrent glioblastoma multiforme or gliosarcoma (NCT03020017). Moreover, it is well-accepted that an increase in our understanding of fundamental signal modulations induced by AuNPs at molecular and cellular levels may help to overcome persistent problems in nanomedicine and lead to AuNPs’ clinical applications. In this review, after a brief excursus on the methods of synthesis of AuNPs and the main applications of AuNPs in oncology, the biological effects of AuNPs on cancer cells and the central role of certain factors, such as the protein corona (PC), the tumor microenvironment (TME) and the surface functionalization, in the contact of AuNPs with cancer cells will be examined in more detail. Finally, the successful application of AuNPs for cancer target discovery will be shortly reviewed.

## 2. Synthesis of AuNPs

Advances in nano-research have allowed the production of different types of AuNPs with well-defined properties, (e.g., size, shape, and surface chemistry) through simple synthetic approaches. The use of gold in colloidal form is attested in antiquity: the most famous artifact containing AuNPs is the Lycurgus cup, which dates to the 5th or 4th century B.C. Although the use of nanometric gold, in this case, was undoubtedly unconscious, certain 17th and 18th century texts reported the existence of colloidal gold, the so-called aurum potabile (drinkable gold) or Purple of Cassius. The first scientific article reporting a synthesis of gold nanoparticles is the famous 1857 Michael Faraday paper [15]. Faraday’s synthesis was based on the chemical reduction of tetrachloroaurate ions (AuCl_4_^−^) with white phosphorous dissolved in carbon disulfide (CS_2_) in a biphasic system. Faraday reports not only the synthesis but also a study on the optical properties of AuNPs. He focused on the colour of the colloid and on the variations in color observed in a dried film of NPs, pioneering the studies on the interactions between spherical particles and on the effect of asymmetric NPs. The synthesis of AuNPs has been widely explored, counting hundreds of papers published in the literature, and many dedicated reviews: a search for the topic “gold nanoparticles” on SciFinder yields more than 70,000 results, with constantly growing numbers and more than 1700 references per year in the years 2019 and 2020. AuNPs are obtained with many different synthetic strategies which can be divided into two categories: top-down and bottom-up syntheses. The former includes a laser ablation [16], cathode sputtering [17], and arc plasma deposition [18]. The latter category includes the “chemical” methods are included, which can all be considered as reduction techniques of different kinds: electrochemical [19], sonochemical [20], thermal [21] or photochemical [22] reduction techniques. The most used (and easiest to manage) synthetic route to obtain AuNPs is chemical reduction. This can take place in water, with all the syntheses derived from Turkevich’s work [23] or in a solvent-mixed phase, with the Brust-Schiffrin approach. Starting from these two “progenitor” papers, a plethora of different synthetic pathways was published in the last decades. Aqueous synthesis is usually preferred for AuNPs designed for use in a medical or biological environment. The Brust–Schiffrin [24] approach yields hydrophobic nanoparticles and requires a further step of functionalization, along with a careful purification from the organic solvent. Aqueous synthesis, on the other hand, yields generally biocompatible products, but is more challenging in terms of shape and size control. In a Turkevich-like synthesis, the three main reagents are a gold source, a reductant, and a stabilizer (capping agent). If the first is typically tetrachloroaurate/tetrachloroauric acid, the reductant and the stabilizer can vary, and a great variety of molecules, polymers and macromolecules have been used in these roles. In 1921 Svedberg [25] listed many reducing agents which were already in use almost a century ago: hydrogen, hydrogen peroxide, hydrogen sulphide, phosphorous, alcohols, glycerine, hydrazines, tartaric acid and sugars, just to name a few. The capping agent is, in some cases, the same molecule used as reductant, as in the case of Turkevich’s synthesis based on the use of citrate. In other cases, the reductant and capping agent are two different molecules, as happens in the synthesis using sodium borohydride as a reductant and citrate as a stabilizer: the use of a stronger reductant leads to a quick reduction and a smaller particle size [26]. Common capping agents for AuNPs, both introduced during the synthesis of after-synthesis functionalization, are thiols [27], amines [28,29] or phosphines [30]. In recent years interest has increased in the green synthesis of noble metal NPs (i.e., gold and silver): this approach is focused on the use of bio-compatible reagents in the role of reductant and stabilizer. An infinite variety of plant extracts have been used for this purpose, including roots, leaves, fruits, bark extracts, etc. [31]. The exact molecule (or macromolecule) involved in the reduction and stabilization processes is often left unknown: most studies lack detail in this area, and the synthesis usually has poor repeatability and size distribution control. To achieve a good compromise between a green synthesis and repeatable and monodisperse nanoparticles, a single molecule or macromolecule of biological origin can be used: chitosan [32], heparin [33], tannic acid [34], and pectin [35], are some examples of this category of molecules. The optical properties of AuNPs are strongly dependent on their size and shape: the position of the surface plasmon band (SPB) in the visible or infrared spectrum is influenced by the shape of the particle and by its aspect ratio (i.e., the length/width ratio, in the simplest case). The effect of an anisotropic shape on the SPB position is much greater than the effect of a size change on a spherical particle. Moreover, anisotropic particles often have more than one SPB in their spectrum, increasing the tuning possibilities of the optical properties. This has led to a great variety of shapes for AuNPs and, consequently, to a plethora of dedicated reviews in the literature [36,37]. A recent review from Ortiz-Castillo et al. contains a useful overview of the seedless syntheses process and of the shapes obtained via this method: nanorods, nanocubes, dodecahedra, nanoprisms and nanostars [37].

## 3. The Application of AuNPs in Oncology

Due to their physical and chemical properties, AuNPs have proven to be successfully used in a wide range of applications in the field of oncology such as in imaging, diagnosis and therapy techniques [13,38,39]. Particularly important physical features are their localized surface plasmon resonance (LSPR), the radioactivity and the high X-ray absorption coefficient [13]. Specifically, the LSPR of AuNPs can be effectively applied in imaging, PTT (photothermal therapy), PDT (photodynamic therapy) and in vitro diagnostics. Meanwhile, the radioactivity of AuNPs can be applied to the imaging and radiographic treatment of tumors and, due to their high atomic number, they have been used for radiotherapy sensitization. Additionally, the chemical properties of AuNPs offer an advantage over many other nanoparticles. In particular, due to their ability to form stable chemical bonds with S- and N-containing groups, the surface of AuNPs can be easily modified and functionalized with a variety of ligands with a specific function, which opens up enormous possibilities for the use of AuNPs as a versatile cancer therapeutic platform, e.g., for cancer targeting and drug delivery [13,38,40]. As shown in Table 1, a simple search of the literature databases (PubMed) shows that huge effort in academic and pre-clinical research is invested in AuNPs associated with chemotherapy, with great emphasis also on targeting and imaging applications. Although the current results of research on AuNPs in cancer field are indeed encouraging, to the best of our knowledge, clinical applications of AuNPs in oncology are almost absent: as reported in the introductory section, only a few examples are in clinical trials, mainly on AuNPs as PTTs and delivery agents [41,42,43,44]. Unfortunately, the toxicity profile of AuNPs and other many factors, including the clearance of the mononuclear phagocytic system (MPS), the renal excretion, the physiological barriers, and the differences between patients decelerate clinical entering of the AuNPs [12,13,45]. The reader interested in the physical and chemical properties of AuNPs exploited in the field of oncology can consult the recent review by Bai et al. [13]. The classes of targeting ligands for targeted anti-cancer therapies with AuNPs were recently described by Goddard et al. [38].

## 4. AuNPs Encounter Cancer Cells

Recent years have witnessed a dramatic increase in the interest of researchers in AuNPs cancer cell interaction and in the molecular mechanisms behind their biological effects, including structural and functional changes in tumor cells. Nevertheless, remain many underestimated and poorly explored factors—present both in vitro and in vivo—that can influence the interaction between AuNPs and cells (not only cancerous but also healthy cells) and can lead to the misunderstanding of experimental results gathered in vitro and impede clinical translation [46,47,48]. The AuNPs-cells interaction is a complex process, and this explains why studying and mapping this interaction is still a challenging task. In the first part of this chapter, we described some factors, in particular the protein corona, the tumor microenvironment and the surface functionalization, involved in the path of AuNPs towards tumor cells, and how they may influence the activity of AuNPs and their recognition by cancer cells. In the second part, we provide a recent update of the major findings on biological effects activated on cancer cells after the interaction with AuNPs.

### 4.1. The Role of the Protein Corona

**Figure 1:**
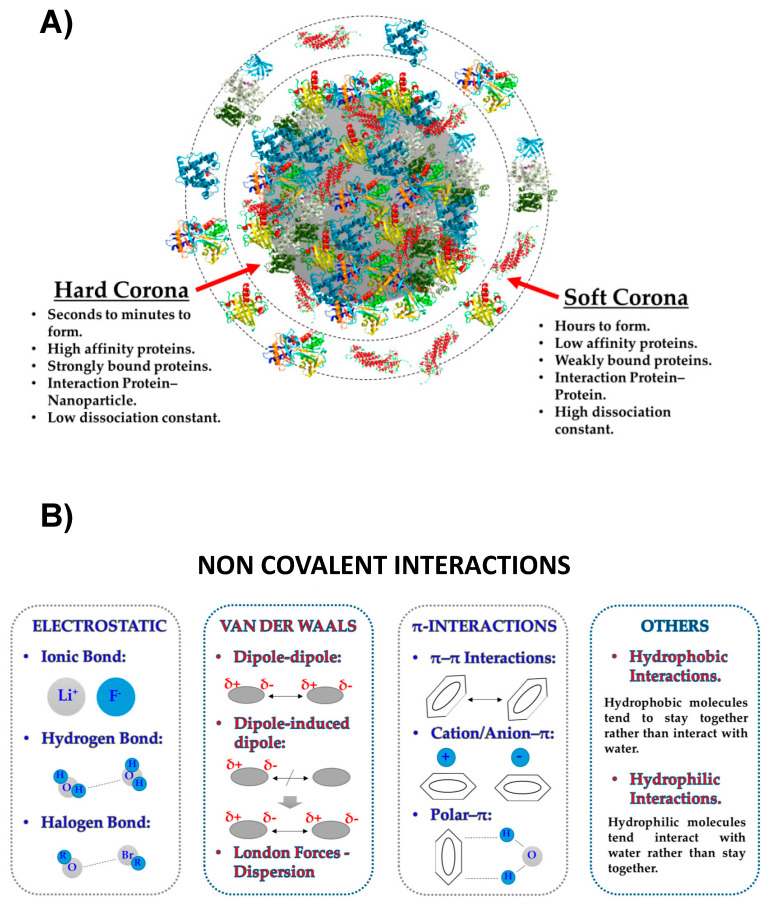
(**A**) Schematic comparison of the main differences between the “hard” corona and “soft” and (**B**) illustration of the different types of non-covalent interactions that could participate in the protein adsorption process adapted with the permission of [52].

To reach the tumor site, AuNPs must pass through several biological barriers, all of which could potentially affect their anti-tumor capabilities, including drug delivery. These include interaction with the proteins when AuNPs are exposed to biological fluids. When AuNPs are introduced into biological systems, their surface immediately interacts with proteins and other biomolecules (like lipids) forming a tightly bound biomolecular (around 20–30 nm thick) corona, termed the protein corona (Figure 1) [49]. Protein interaction with NPs surface is a dynamic and complex process, driven by a minimization of NP high surface free energy in which there is continuous absorption/desorption of different molecules that compete for the NP available surface [50,51]. The protein corona is made up of both hard and soft layers, depending on the affinity of the proteins for the nanoparticle surface. The “hard” corona represents the inner layer of high-affinity proteins that usually remain irreversibly bound to the NP’s surface, while the “soft” corona is the outer layer of weakly bound proteins that exhibit a shorter lifetime [52]. In recent decades many studies have tried to explain the protein corona formation [53,54]. According to the Vroman effect, this process seems to be characterized in the initial step by the attachment of fast exchange protein with weak affinity (the soft corona) to NP surfaces followed by a series of movements and/or rearrangements toward irreversible protein adsorption through their displacement and the attachment of slow exchange proteins with higher affinities for NP surfaces, the hard corona, to make a more stable, strong and ultimately irreversible attachment to the surface [55]. Principally, hydrophobic, and electrostatic interactions, and hydrogen bonds, are involved in the mechanism by which proteins attach to surfaces [56]. The resulting NPs coated by PC gain a new biological identity, and this corona is what is ultimately “seen” by cells. As a result of the PC layer the behaviors of NPs—interaction with a living system, such as cellular uptake, biodistribution, signaling, circulation lifetime and toxicity—can be modified [52,57]. Thus, the formation of the protein corona may be disadvantageous for most in vivo applications of AuNPs. Once the AuNPs are placed in a biological environment, corona proteins can significantly reduce or eliminate the active targeting capability of AuNPs, as the PC may sterically hinder the pre-coupled targeting ligands which may then fail to recognize their specific receptors or relevant targets pathways on cells [58]. In addition to these modifications, the surface targeting ligands may lose or change their structure after interaction with the PC. This can comprise the distribution of AuNPs in the body, leading for example to unintended accumulation in non-target tissues: an effect which can render self-proteins immunogenic, thereby eliciting an autoimmune response [59,60]. The amount, thickness, and composition of the PC are influenced by different factors, including the physicochemical property of AuNPs, such as the size [61], the hydrophilicity and hydrophobicity [62] and the surface chemistry [63]. The size of AuNPs seems to be one of the most relevant parameters. The particle size, and the surface curvature influence the amount of absorbed protein and, to a lesser extent, the identity of these bound proteins [51,64,65]. In the past, most studies have focused on spherical AuNPs [64,66] but more recent publications report protein corona studies on AuNPs of different geometries, such as nanorods [67,68] and nanostars [69]. Other contributing factors in PC nature are the experimental parameters, such as the incubation conditions and the characteristics of the surrounding environment: i.e., ionic strength of buffer media, pH values, the presence or concentration of plasma proteins as additional nutrient sources [70,71,72]. All these parameters can alter the features of AuNPs in solution, provide an inaccurate assessment of AuNPs toxicity and efficacy and can cause different biological responses [56,70]. In summary, since the biological identity of the NPs is ultimately determined by the PC, all these factors need to be considered by researchers. Numerous efforts have been made in the past to chemically modify the AuNPs, for example deploying various synthetic coatings (i.e., polyethylene-glycol, PEG) and natural polymeric coatings (i.e., chitosan, etc.) [73,74,75], to maintain the physicochemical properties and functional integrity of AuNPs following exposure to biological systems and to prevent PC formation and the agglomeration and targeting of NPs by the immune and reticuloendothelial systems (RES), which are responsible for NPs clearance from the bloodstream [76].

### 4.2. The Tumor Microenvironment

The TME is another significant barrier to AuNP deployment. The TME is a complex tissue containing several cell types, such as cancer cells (CCs), cancer associated fibroblasts (CAFs), and endothelial cells (ECs). The TME also regulates cancer cell behaviors, persistently provides nutrition for cancer progression and metastasis, and has a central role in therapy resistance [45,56,77]. The principle of action using nanoparticles for cancer drug delivery is commonly based on their accumulation in solid tumor tissue through a process known as the enhanced permeability and retention effect (EPR). This phenomenon is made possible by the increased leakiness of the tumor vasculature, compared to the normal state, which allows for NPs to leave the bloodstream and traverse through the gaps in the endothelial lining of the vessels to enter the tumor site [78]. This phenomenon, however, can also be a hindrance: it does not enable uniform delivery of these particles to all regions of the tumor in sufficient quantities [79]. This heterogeneous distribution of therapeutics is a result of physiological feature presented by the TME. TME is quite different from the microenvironment of normal tissues. The pathophysiological state of TME consists of aberrant characteristics, including hypoxia, acidosis, elevated interstitial fluid pressure, abnormal interstitial matrix, and heterogeneity of vasculature [45,80]. The heterogeneity of the tumor vasculature, which is differentiated for vessel maturity, perfusion, density, and pore size, not only forms abnormally, inducing proliferative, hypoxic, and necrotic tumor tissue regions but also has an important role in determining the EPR effect of NPs delivery and accumulation into the tumor tissue. The inherent abnormalities of the TME represent a barrier to the effective transport processes of small molecules as oxygen [81] and nanomedicines [80,82]. In general, a great deal of effort has been made to overcome these barriers and to realize the promise of nanomedicine. For instance, attempts have been made to modify the TME with the use of antiangiogenic and matrix-modifying agents [82] or to redesign NPs (their size, shape and charge and all relevant physicochemical features) to enhance the transport processes into tumor tissues [83,84]. A very popular strategy to improve the distribution and delivery of nanomedicine to solid tumors employs nanoparticle systems that are able to release therapeutic agents in response to the TME conditions (pH, temperature, enzyme activity, redox) or to an external stimulus (light, ultrasound, heat, electric or magnetic fields) [82,85,86]. In this scenario, ultrasmall AuNPs (less than 10 nm in diameter) have gained particular attention due to their small size, lower systemic toxicity, faster kidney clearance rate and higher tumor accumulation [39,40]. This type of AuNPs was recently employed Ding et al. to design and synthesize TME-responsive multifunctional peptide-modified ultrasmall AuNPs (diameter smaller than 5 nm) to enhance the accumulation of anticancer agents tumor sites in vivo and to amplify the cancer radiotherapy [87]. A first attempt at the development of an approach to predict the efficacy of AuNPs based therapy by using patient-–tumor-specific characteristics, such as proliferative index and vascular density was covered in a recent paper by Miller and Frieboes [88]. These authors used a computational simulation to evaluate the role of vascular density-induced heterogeneity on the distribution of 3-layered AuNPs in tumor tissue and the associated drug release, providing a means to quantify the effect of tumor tissue vascular density on the response to nanotherapy and, at the same time, to help define nanoparticle formulation and delivery methods. For more information on the various types of alternative strategies for enhanced nanoparticle delivery to solid tumors, a thorough review by Izci et al. can be consulted [85]. In short, the careful design of AuNPs formulations may overcome barriers posed by the TME to obtain better cancer treatment.

### 4.3. The Importance of Surface Functionalization

A large body of current literature has focused on the complex molecular mechanisms governing the interactions of AuNPs with biological systems and the subsequent activation of signal transduction pathways. The cell response begins when the AuNPs bind to cellular receptors (specific interaction) and the cell membrane (nonspecific interaction), followed by internalization. The majority of AuNPs are taken up by cells, including cancer cells, through receptor-mediated endocytosis (RME) process [89,90]. This is a mechanism that internalizes cargo in transport vesicles derived from a plasma membrane [91]. The endocytosis process is followed by NP accumulation and distribution in different types of endocytic vesicles. Once inside the endocytic vesicles, AuNPs are carried to specific and specialized intracellular sorting/trafficking organelles [92,93]. It is well-documented that AuNP characteristics—e.g., charge, surface functionalization, particle size and shape, time and exposure time—are critical parameters for their interaction with cancer cells and their delivery to organelles [56,94,95]. The most important characteristics of nanoproducts to be suitable for biomedical purposes, such as cancer therapy, are their biocompatibility and uptake capability in target cells [96]. The accumulation of AuNPs can occur through two different mechanisms: passive or active targeting [38,97]. The untargeted AuNPs (as-synthesized, i.e., unmodified or uncapped) display a passive targeting towards tumors. This is achieved through the EPR effect which allows for the accumulation of AuNPs in solid tumors and/or metastatic sites through the exploitation of the physical properties of AuNPs including size, shape, and surface charge [78]. These properties are, undoubtedly, advantageous for the targeting of cancer cells but there are also concerns related to the internalization of nanoparticles in healthy cells [98] and their non-specific accumulation within various tissues and organs in the body, especially in the reticuloendothelial system organs [99,100]. In recent years, the urgent calls to drive forward personalized medicine have been focused on the active targeting of AuNPs for tumor diagnosis and treatment, both to increase retention rates in tumor tissues and to reduce accumulation in other tissues. Active targeting involves the functionalization of AuNP surfaces by targeting moieties, which are specific to a wide range of overexpressed surface receptors in the cancer cells, such as EGFR (epidermal growth factor receptor), human epidermal growth factor receptor-2 (HER-2) and CD44 [38,101] and molecules in the TME [102,103]. As extensively documented by many reviews [93,97], the functionalization of AuNPs with a plethora of targeting moieties—through electrostatic adsorption or with covalent bonds by using crosslinkers with -SH or -NH_2_ groups which are able to react with the metal [97,104,105]—have been explored to support the active targeting of cancer cells and a number of very promising strategies for the selective delivery of chemotherapy drugs or molecules that must be released in the tumor cells have been developed [38,106]. To date, proteins [107], peptides [108], aptamer [109], lectins [110], carbohydrates [111], small molecules [112] and antibodies [113] have been exploited as targeting ligands. Among antibodies, two clinically approved monoclonal antibodies, cetuximab (anti-EGFR) and trastuzumab (anti-HER-2) were used to functionalize AuNPs for enhanced cancer radiotherapy or to depict and quantify high and low tumoral surface marker expression [114,115]. As the large size of antibodies can potentially inhibit their penetration into tumors, antibody fragments or nano-bodies (smaller than antibody fragments) have recently attracted great attention as viable targeting moieties [116,117]. The surface modification of AuNPs with functional groups (including acetyl, zwitterionic, carboxyl) can also enhance the passive uptake of AuNPs by masking them from phagocytic cells or by increasing their interaction with cancer cells and their penetration into their cell membrane [94,105]. Peter et al. found that positively charged AuNPs (N-trimethoxysilypropyl-N, N, N-trimethylammonium-functionalized AuNPs) were able to penetrate cancer cells more effectively than negatively charged AuNPs. The overexpression of sialic acid-rich regions in cancer calls [118] can contribute to the creation of a negative charge on cell surfaces which, in turn, increases their interaction with positive charged AuNPs [119]. Many other types of molecules, (i.e., rifampicin, cytosine-phosphate-guanine oligonucleotides, and amphiphilic drugs such the small molecule Toll-like receptor agonist resiquimod (R848)) have recently been used to functionalize AuNPs in order to increase their target specificity, cellular uptake rate, immune stimulation and therapeutic properties [120,121,122]. Despite a vast number of scientific papers confirming the important role of the surface functionalization with specific ligands as strategy to improve biocompatibility both in vitro and in vivo, and so to allow for the development of high precision therapy and/or diagnosis, a recent review by Goddard et al. has highlighted that each targeting ligand has both benefits and drawback and argued that it is necessary to make comparisons between different targeting modalities for the same receptor to establish the most effective targeting approach [38].

### 4.4. The Main Biological Effects

**Figure 2:**
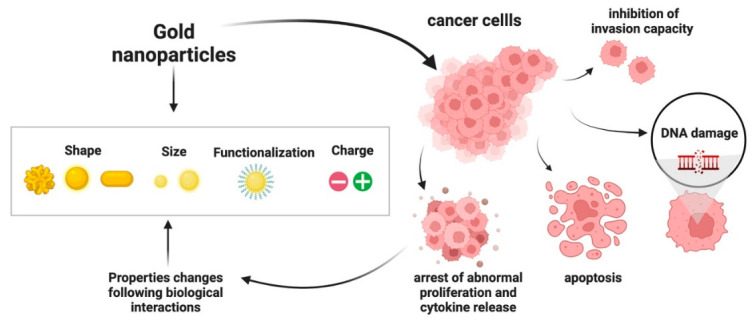
Summary of biological effects by AuNPs on cancer cells created with BioRender.com.

In general, AuNPs have antineoplastic biological activity due to their extrinsic functionalization or activation by ligands with specific functions, but they also may have an intrinsic antitumor capacity [123,124,125], inhibiting, for instance, the growth of cancer cells through the abrogation of MAPK-signaling and preventing epithelial plasticity (EMT) [124]. The AuNPs size plays a pivotal role in the intrinsic therapeutic effect [123,125,126]. In line with previous research, Wu et al. found that smaller AuNPs (5 nm) inhibited cell proliferation and invasion of gastric cancer cells, but no effect was found after the treatment with 10-, 20- and 40-nm AuNPs groups [123]. Most of the currently investigated AuNPs induced a range of adverse effects in various types of cancer cells (Figure 2) [56,127,128,129,130]. As previously summarized by Wang et al. [56] different cancer lines showed significant levels of susceptibility to the toxicity of differently shaped and size AuNPs. Most studies report the cellular damage caused by spherical and rod-shaped AuNPs. There are, conversely, very few reports on the biological impact of branched AuNPs, such as gold nanostars [131,132], which can, due to their large surface/volume ratio, be used for photothermal ablation with near-infrared light [133]. Several studies have revealed that the effect of AuNPs is impacted by excessive reactive oxygen species (ROS) production and subsequent oxidative stress [134,135]. The treatment of cancer cells with AuNPs can also led to the marked induction of intrinsic and extrinsic apoptosis-related pathways [113,136,137,138], and to metabolic changes that can cause oxidative stress [139]. AuNPs can also reprogram the pancreatic TME and inhibit tumor growth by disrupting the bidirectional communication in the TME via alteration of the cell secretome [140]. Kodiha et al. demonstrated that toxicity of AuNPs with different morphologies correlated with changes in nuclear organization and function [141]. In another work, the same authors also reported that nucleoli were particularly prone to AuNPs-induced injury, reporting that B23/nucleophosmin, a nucleolar protein that can function as a tumor suppressor or proto-oncogene, was significantly affected by exposure to AuNPs [142]. Moreover, AuNPs reduced the rate of cell growth and delayed the cell cycle in a dose-dependent manner in different cancer cell lines [99]. AuNPs enter the cell nucleus, where they likely exert their effects on DNA [143,144]. Properly conjugating AuNPs with specific peptides can enable them to be selectively transported to the nuclei of cancer cells, disturbing the division of cancer cells by DNA damage, cytokines arrest and apoptosis activation [145,146,147,148]. It has also been reported that AuNPs are able to amplify the formation of strand breaks upon exposure to ionizing radiation [149]. In this context, particularly interesting are the ultrasmall AuNPs. A very recent study has demonstrated that peptide-modified ultrasmall AuNPs lead to selective uptake by cancer cells in vitro and accumulation to tumor sites in vivo, increasing the effective DNA damage upon X-ray irradiation [87]. The research work of Taggart et al. identified the biological response mechanisms of thiol-coated AuNPs (9-nm) in combination with an ionising radiation [150]. Using N-acetyl-cysteine, they found that irradiation in the presence of AuNPs results in the interaction of AuNPs with the cell membrane protein disulfide isomerase (PDI) which, in turn, led to an overall disruption of thiol balance within the cell which significantly increased cell killing. Overall, although the biological effects of AuNP may be different, depending on cell type and physicochemical characteristics, in vitro results can provide relevant insights prior to in vivo application of AuNPs.

## 5. Challenges of the Application of AuNPs in Cancer Treatment

Although numerous in vitro and in vivo studies demonstrate the enormous potential of gold-based nanomedicines in the field of oncology, some critical issues slow down their clinical application. One of the biggest challenges concerns the toxicity of AuNPs. AuNPs have generally been regarded as inert, like bulk gold, but they may have inherent chemical toxicity, particularly in relation to their intrinsic properties, (i.e., size, shape, charge, and composition) [99,151,152,153]. Furthermore, AuNPs are not biodegradable and off-target distribution could cause chronic and lethal health effects [99]. Therefore, there is an urgent need for more in-depth studies to clarify and research the factors influencing the pharmacokinetics, biodistribution and in vivo toxicity of AuNPs. However, determining which parameters are most important in the in vivo toxicity and biodistribution of AuNPs is very challenging for many reasons: (i) the available publications differ widely in experimental methods and conditions (differences in exposure time and dose [12] and route of administration [151,154]; (ii) species-specific differences in reactions and sensitivity [155]; (iii) the limitations of current pre-clinical models (testing the toxicity of AuNPs in conventional 2D models may not accurately reflect the AuNPs-cell interaction) [156]. Another challenge concerns ligands used for the functionalization of AuNPs. As pointed out in the previous paragraphs, functionalizing the surface of AuNPs with biocompatible and cancer-specific molecules can direct and limit their effect to cancer cells, thus reducing toxicity effects [12]. However, prior to their application as a targeting ligand, problems related to their purification, immunogenicity and toxicity must be addressed [157]. AuNPs can be efficiently produced chemically, but the main risk is the formation of by-products harmful to human health and the environment [158]. New and safe synthesis methods are therefore indispensable. The development of green synthetic routes using natural materials, such as plant extract, bacteria, and fungi, is a fascinating field of research due to its effectiveness in synthesizing environmentally friendly AuNPs and its cost-effectiveness, which will open up a new route for the production of biologically active, non-toxic AuNPs [130,159]. Lastly, it is highly hoped that systematic studies [160] and a standardized approach for physicochemical characterization and pre-clinical testing will undoubtedly help to clarify the AuNPs-cell biological interaction and predict the impact of AuNPs on human health, prior to clinical translation.

## 6. Exploiting the Contact of AuNPs with Cancer Cells Proteins for the Discovery of New Molecular Targets

AuNPs have been widely studied for their potential in disease therapeutics as targeting agents, drug delivery vehicles and as therapeutic agents themselves [161]. As described in Section 4.1, the presence of AuNPs in the biological system and interaction with various biological proteins leads to the protein corona development. Recently there has been extensive work on deciphering the interactions between proteins and nanoparticles in various biofluids and their biophysical properties, in the context of drug delivery and therapeutics. Emerging studies have demonstrated that the identification of PC content can provide a unique insight into the biological functions of the AuNPs, including their biodistribution, clearance and potential toxicity [6,162]. It is also clear that the physicochemical properties of AuNPs (size, charge, surface functional groups, shape) significantly influence protein adsorption and so the PC composition ad patterns [162]. Taking this consideration into account, an idea has been evolving among researchers to employ nanoparticles as a unique platform to sequester and enrich low abundance proteins and to identify new therapeutic targets for diseases such as cancer: a field of research that is known as nanoproteomics [163,164]. NPs have been introduced to the field of proteomics and have been employed to improve the specificity, reproducibility, and robustness of the current proteomic methods by manipulating individual proteins [163]. Indeed, the large surface to volume ratio of nanomaterials simplifies mass transfer and increases efficiency of separation for various peptides whilst reducing assay time and sample consumption, and the functionalization of nanomaterial ‘surface, including polymer NPs, AuNPs, silica NPs, fullerenes and carbon nanotubes facilitate the specific separation of peptides [165]. In this way, different researchers have identified novel molecular targets after the analysis of the PC formed around nanomaterials from both healthy and pathological samples (serum/plasma samples) [166,167,168], tissues lysates [169] and cell lysates [170]. In the cancer field, a limited number of works have demonstrated the effectiveness of AuNPs (in particular the spherical ones) for improving proteomic analysis and cancer target identification (Table 2). Arvizo et al. characterized the “fingerprint” of the protein corona developed around the surface-functionalized AuNPs (positively and negatively charged) upon incubating from normal and malignant ovarian cell lysates. Using a combined approach (proteomics, bioinformatics, and nanotechnology) they successfully enriched and identified hepatoma-derived growth factor (HDGF) in the corona of positively charged AuNPs after incubated with cancer cell lysates. Further, they confirmed the role of HDGF as a new target for cancer treatment through silencing of HDGF, which inhibited the proliferation of ovarian cancer cells [170]. The examination of PC surrounding AuNPs can be used to discover novel biomarkers starting directly from human serum, and comprehensively analyze the enriched proteoforms by mass spectroscopy. In this context, recent studies from De Pilar and colleagues clearly demonstrated that the analysis of AuNPs (10.02 ± 0.91 nm) surrounding PC, following incubation with human serum from triple-negative cancer (TNBC) patients, allowed for the identification of novel biomarkers (prognostic and diagnostic) of TNBC [167]. Recently, another group characterized and identified key proteins implicated in thyroid cancer metabolic reprogramming, cancer progression, and metastasis by analyzing protein extracts from thyroid tissue samples (health and cancerous) using a nanoparticle-assisted proteomics approach based on AuNP-, AgNP- (silver nanoparticles and FeNP- (magnetic nanoparticles) combined with shotgun LC-MS/MS [169]. Two potential biomarkers of follicular thyroid adenomas (FTA) were identified in the PC of AuNPs: the aldo-keto reductase family 1 member C1 (AKR1C1) and the heterogeneous nuclear ribonucleoprotein C-like 2 (HNRNPCL2), both proteins linked to cancer progression and malignant transformation [171,172], which could be possible molecular targets and, at the same time, targeted for maximum therapeutic benefit in the treatment of thyroid cancer. These promising AuNPs-based approaches suggest that they could be applied in the near future to identify new potential targets for both therapy and diagnosis of a wide range of cancers. Nevertheless, we must deal with the limitations of the technology (i.e., proteins or peptides loss during proteomic sample processing) and fill the remaining gaps in our comprehension of the molecular mechanisms of AuNPs-cell interactions [163,173]. Improved nanoproteomic technologies, together with the integration of the data obtained with genome and transcriptome results, may help us reveal new molecular targets and enable a better understanding of the molecular basis underlying cellular heterogeneity in both normal and pathological tissues.

## 7. Conclusions

In recent decades, the astounding advances in nanotechnology have led to the development of a wide, and heterogeneous, range of AuNP-based platforms for the diagnosis and treatment of oncological diseases. There is no doubt that the physicochemical properties, (i.e., size, shape, charge, and/or surface molecules) of AuNPs are responsible for different biological responses in cancer and it is equally clear that both cancer cell type and tissue microenvironment play a central role in the cellular uptake and cytotoxicity of AuNPs. Similarly, AuNP surface modification is a powerful instrument to enhance uptake by cancer cells and biocompatibility. Generally, there is a strong need for more and more detailed studies of the mechanisms and the biological effects of AuNPs. Greater understanding in this area can drive forward the rational design of AuNPs which, with the support of in vivo validation, can reduce unnecessary animal experiments and lower unsuccessful outcomes. Ultimately, we have highlighted the role of AuNPs as a useful tool for the enrichment and detection of low abundance proteins and as a unique approach to discovering new diagnostic and therapeutic targets in cancer diseases. We also note, in this context, that the use of more consistent and standardized techniques across studies would be of great benefit and would undoubtedly enhance our understanding of the potential benefits of this approach to AuNPs in the field of tumor target discovery.

## Figures and Tables

**Table 1:** Application of AuNPs in oncology. Search was performed on 28 May 2022 using the PubMed database.

Search String	Number of Results (www.ncbi.nlm.nih.gov/pubmed)
Gold nanoparticles AND chemotherapy	5053
Gold nanoparticles AND photothermal therapy AND cancer	1346
Gold nanoparticles AND radiotherapy AND cancer	596
Gold nanoparticles AND imaging AND cancer	2540
Gold nanoparticles AND targeting AND cancer	2984

**Table 2:** A summary of literature findings using proteomic approaches based of AuNPs.

Shape	Size (nm)	Coating	Charge	Target Type	AuNPs Incubation with	Approach	Identified Target	Ref.
sphere	10	TTMA or TCOOH	negative or positive	human ovarian cancer cell lines (OV167, A2780)	cell lysates	nanoLC-ESI-MS/MS	HDGF	[170]
sphere	20	citrate	negative	human ovarian cancer cell line (A2780)	cell lysates	nanoLC-MS/MS	PPA1, SMNDC1, and PI15	[174]
sphere	~30	citrate-BSA	negative	human colon adenocarcinoma cell line (Caco-2)	cell lysates	LC-MS/MS	new pathway from endosomes to secretory vesicles	[175]
sphere	~7.5	citrate	negative	neoplastic thyroid	proteins extracted from human tissue sections	LC-MS/MS	Proteins implicated in thyroid Cancer progression and metastasis	[169]
sphere	~10	citrate	negative	TNBC	human serum	LC-MS/MS quantification by SWATH acquisition	several breast cancer-specific markers	[167]
sphere	~13	citrate	negative	different human breast cancer intrinsic subtypes (LA, LB−, LB+, HER-2+ and TNBC)	human serum	LC-MS/MS quantification by SWATH acquisition	profile of blood coagulation proteins	[176]

**Abbreviations:** TTMA, thioalkyl tetra(ethyleneglycol)ated trimethylammonium; TCOOH, carboxylate ligands; nanoLC-ESI-MS/MS, nano-flow liquid chromatography electrospray tandem mass spectrometry; nanoLC-MS/MS, nanoscale liquid chromatography coupled to tandem mass spectrometry; PPA1, Pyrophosphatase (Inorganic)1; SMNDC1 (survival motor neuron domain containing 1; PI15, Peptidase Inhibitor 15; PVP, poly-N-vinylpyrrolidone; LC-MS/MS, liquid chromatography tandem mass spectrometry; BSA, bovine serum albumin; SWATH-MS, sequential window acquisition of all theoretical mass spectra; triple negative breast cancer, TNBC; luminal A, LA; luminal B negative, LB−; luminal B positive, LB+; HER-2 positive HER-2+.

## Data Availability

Not applicable.
